# A Prospective Pilot Study to Identify a Myocarditis Cohort who may Safely Resume Sports Activities 3 Months after Diagnosis

**DOI:** 10.1007/s12265-020-09983-6

**Published:** 2020-05-04

**Authors:** D. Patriki, N. Baltensperger, J. Berg, L. T. Cooper, C. K. Kissel, J. Kottwitz, M. Lovrinovic, R. Manka, F. Scherff, C. Schmied, F. C. Tanner, T. F. Luescher, Bettina Heidecker

**Affiliations:** 1 Cardiology, University Heart Center, Zurich, Switzerland; 2 Mayo Clinic, Jacksonville, FL USA; 3 Center for Molecular Cardiology, University of Zurich, Zurich, Switzerland; 4 Imperial College and Royal Brompton & Harefield Hospital, London, UK; 5 Charite Universitätsmedizin Berlin, Campus Benjamin Franklin, Germany, Hindenburgdamm 30, 12203 Berlin, Germany

**Keywords:** Myocarditis, Sudden cardiac death, Magnetic resonance imaging, Exercise, Arrhythmia

## Abstract

International cardiovascular society recommendations to return to sports activities following acute myocarditis are based on expert consensus in the absence of prospective studies. We prospectively enrolled 30 patients with newly diagnosed myocarditis based on clinical parameters, laboratory measurements and cardiac magnetic resonance imaging with mildly reduced or preserved left ventricular ejection fraction (LVEF) with a follow-up of 12 months. Cessation of physical activity was recommended for 3 months. The average age was 35 (19–80) years with 73% male patients. One case of non-sustained ventricular tachycardia was recorded during 48-h-Holter electrocardiogram. Except for this case, all patients were allowed to resume physical exercise after 3 months. At 6- (*n* = 26) and 12-month (*n* = 19) follow-up neither cardiac events nor worsening LVEF were recorded. The risk of cardiac events at 1 year after diagnosis of myocarditis appears to be low after resumption of exercise after 3 months among patients who recover from acute myocarditis.

Athletes appear to be at special risk for sudden cardiac death following acute myocarditis, possibly because intense physical exercise increases viral replication and suppresses the immune system. In turn, this may lead to enhanced susceptibility for upper respiratory infections [1]. It has been hypothesized that the inflammatory process accompanying a virus-associated myocarditis also involves conduction tissue in ways that can lead to potentially lethal arrhythmias [2]. As a result of these findings, physical activity is contraindicated for a period of 3 to 6 months after a diagnosis of myocarditis [3, 4]. Importantly, the recommendation to refrain from physical activity following an episode of virus-associated myocarditis has not been critically examined and the optimal duration of exercise cessation remains unknown.

To address the question of exercise following myocarditis, we prospectively evaluated the safety of early resumption of exercise 3 months following diagnosis with myocarditis in asymptomatic patients without arrhythmias on 48-h-Holter electrocardiogram (ECG) monitoring or stress testing with normal or recovered left ventricular ejection fraction (LVEF). Outcomes included adverse cardiovascular events, defined as persistent or malignant arrhythmias, worsening LVEF, hospitalizations for heart failure, and death after resumption of exercise at 3-month until 12-month follow-up.

Myocarditis was defined according to ESC diagnostic criteria, including: Clinical presentation with dyspnea, chest pain, palpitations, elevated high sensitivity troponin T (TnT-hs) (> 14 ng/l), angiographic exclusion of obstructive coronary artery disease (grade of stenosis <50%), and characteristic features on cardiac magnetic resonance imaging (CMR) [5]. Subjects were advised to avoid strenuous exercise for 3 months. In order to minimize the risk for adverse events in this first prospective trial, patients with severely reduced LVEF (<35%) at the time of enrolment were not included in this study.

At 3, 6 and 12 months follow-up subjects received a cardiovascular examination including history and physical exam, laboratory testing of C-reactive protein (CRP; normal range < 5 mg/l), TnT-hs (normal range < 14 ng/l), myoglobin (Mb; normal range 28–72 μg/l), creatine kinase (CK) (normal range < 190 U/l), N-Terminal B-type natriuretic peptide (NT-proBNP) (normal range < 125 pg/ml), leukocytes (Lc; normal range 3–9.6 G/l), resting 12-lead ECG, 48-h-Holter ECG, exercise stress testing with ramp protocol, and echocardiography. CMR was repeated as part of the 3 months follow-up.

Our population consisted of 30 patients with a median age of 35 years (range: 19–80 years) of whom 8 (27%) were women and 22 (73%) were men. Baseline characteristics are presented in Table 1. Due to loss of follow-up, 26 patients presented for 6 months follow-up and 19 patients presented for their 12-month visit (Fig. 1). All patients who did not present for evaluation after 6 or 12 months were contacted by telephone call. Overall, no cardiac events were recorded during the time period of 12 months after symptom onset.

**Table 1:** Baseline characteristics of all patients with myocarditis.

Baseline characteristics all cases of myocarditis	Value (*n* = 30)
Male Sex, n (%)	22 (73)
Median age (IQR)	32 (22–42)
Mean BMI kg/m^2^, (±SD)	26.7 (±4.6)
aHTN, n (%)	3 (10)
HLD, n (%)	3 (10)
DM, n (%)	1 (3)
History of smoking, n (%)	10 (33)

Abbreviations: aHTN = arterial hypertension; BMI = body mass index; CAD = coronary artery disease; DM = diabetes mellitus; HLD = hyperlipidemia;

**Fig. 1:**
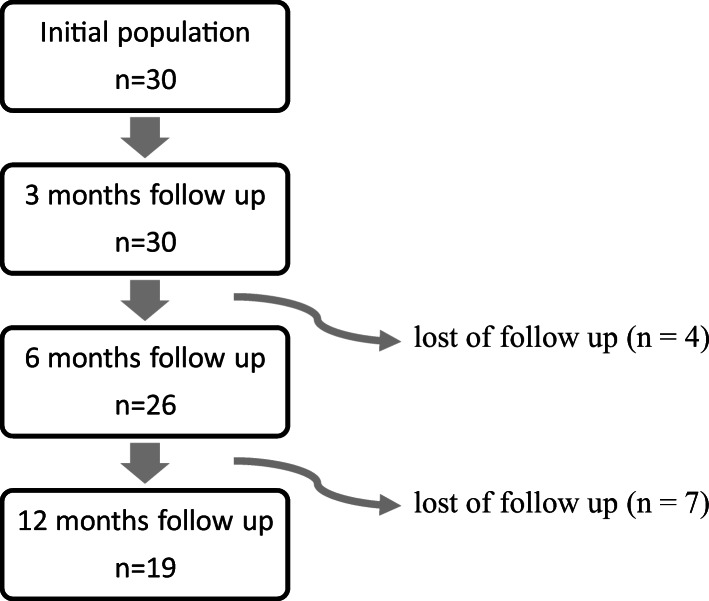
Flowchart of our population

Initially, all patients showed patchy or diffuse midwall late gadolinium enhancement (LGE) patterns. In 16 (53%) cases additional edema was present in T2 weighted images.

After 3 months visually assessed extent of LGE decreased in all patients. No edema was detected on repeat CMRs and 7 (24%) patients showed complete remission of LGE.

At presentation, the averaged LVEF was 58% (± 7.4). Three patients revealed a mid-range reduced LVEF. During the follow-up, one remained stable while others normalized.

During follow-up stress testing, neither significant ECG changes nor pathological symptoms were provoked.

After 3 months, 48-h-Holter ECG showed episodes of non-sustained ventricular tachycardia in one patient. As a consequence, physical exercise was discouraged in this case until further investigations at 6-month follow-up. This 19-year-old man initially presented with severe dyspnoea NYHA III; however, only mild angina-like symptoms were present. The LVEF was stable with elevated CRP 12 mg/l, TnT-hs 276 ng/l, Mb 41 µg/l, CK 213 U/l, and normal NT-proBNP 158 ng/l and Lc 6.95 G/l. After 6 months, all elevated biomarkers normalized and 48-h-Holter ECG as well stress testing showed no signs of arrhythmias. On CMR, initial LGE declined after 3 months and edema on T2-weighted images disappeared. Results from repeated laboratory testing are demonstrated in Table 2.

**Table 2:** Overall findings at time of onset, 3 and 6-month follow-up.

Baseline characteristics all cases of myocarditis	Time of symptom onset	3-month follow-up	6-month follow-up	12-month follow-up	*P* Value
CMR/Echocardiography	n = 30	n = 30	n = 26	n = 19	
Mean LVEF (±SD)	58 (±7.44)	59 (±6)	59.4 (±4.8)	58 (±5.3)	NS
Resting ECG	n = 30	n = 30	n = 26	n = 19	
Number of malignant arrhythmias	0	1	0	0	NS
Number of benign arrhythmias	8	6	4	4	NS
48-h-Holter ECG	n = 0	n = 30	n = 26	n = 19	
Number of malignant arrhythmias	/	1	0	0	NS
Number of benign arrhythmias	/	6	6	4	NS
Median APC/QRS % (IQR)	/	0.0172 (0.008–0-047)	0.036 (±0.055%)	0.034 (0.002–0.06	NS
Median PVC/QRS % (IQR)	/	0.008 (0–0.0029)	0. 16% (±0.782%)	0.0026 (0–0.02)	NS
Exercise stress test	n = 0	n = 29	n = 25	n = 19	
Mean performance, watts (±SD)	/	190 (±67)	195 (±75)	206 (±75)	NS
Mean % of predicted maximum watt	/	95% (±27)	98 (±26)	105 (±26)	NS
Number of malignant arrhythmias	/	0	0	0	NS
Laboratory results (median)	n = 30	n = 30	n = 26	n = 19	
Mb, ng/l (IQR)	32 (0–111)	27 (0–40)	22.7 (±31.1)	27 (0–43)	0.011
TnT-hs, ng/l (IQR)	350 (88–1104)	2.5 (0–7)	4.4 (±7.5)	0 (0–7)	<0.001
CK, U/l (IQR)	238 (128–587)	97 (66–156)	130.3 (±63)	119 (75–193)	0.001
NT-proBNP, ng/l (IQR)	251 (45–676)	30 (11–44)	63 (±84)	38 (13–121)	0.05
CRP, mg/l (IQR)	21 (3–66)	0.7 (0.4–2.6)	1.6 (±1.8)	0.9 (0.5–1.6)	<0.001
Lc, G/L (IQR)	7 (6–9)	6.8 (5.9–7.2)	5.9 (±2.3)	6 (5.6–7.6)	NS

Abbreviations: CMR = cardiac magnetic resonance imaging; SD = standard deviation; ECG = electrocardiogram; APC = atrial premature contraction; TnT-hs = high sensitivity troponin T, CK = creatine kinase; CRP = C-reactive protein; Lc = leukocytes; LVEF = left ventricular ejection fraction; Mb = myoglobin; NT-proBNP = NT-pro brain natriuretic peptide; NS = not significant; IQR = interquartile range; PVC = premature ventricular contraction

In a first attempt we evaluated early resumption of physical activity after 3 months in a small cohort of patients who recover from acute myocarditis. This allowed us to identify patients without clinical abnormalities such as increasing levels of TnT-hs, arrhythmias in resting 12-lead ECG or 48-h-Holter ECG, worsening of maximum power on exercise stress testing or worsening LVEF as potentially good candidates for early resumptions of exercise.

Due to the small sample size, the statistical power of this study is limited. Given the numerous beneficial effects of physical activity, further research is necessary to determine the right timing for resumption of exercise after the onset of myocarditis.

## Abbreviations

LVEF: left ventricular ejection fraction
CMR: cardiac magnetic resonance imaging
ECG: electrocardiogram
LGE: late gadolinium enhancement
SD: standard deviation
TnT-hs: high sensitivity troponin T
CK: creatine kinase
CRP: c-reactive protein
Lc: leukocytes
Mb: myoglobin
NT-proBNP: n terminal pro brain natriuretic peptide
NT: pro brain natriuretic peptide
NS: not significant
IQR: interquartile range
AHTN: arterial hypertension
BMI: body mass index
CAD: coronary artery disease
DM: diabetes mellitus
HLD: hyperlipidemia
ECG: electrocardiogram
LVEF: left ventricular ejection fraction
CMR: cardiac magnetic resonance imaging

## References

[1] Finocchiaro G, Papadakis M, Robertus J-L, Dhutia H, Steriotis AK, Tome M (2016). Etiology of sudden death in sports. Journal of the American College of Cardiology.

[2] Frustaci A, Petrosillo N, Ippolito G, Chimenti C (2016). Transitory ventricular tachycardia associated with influenza a infection of cardiac conduction tissue. Infection.

[3] Pelliccia A, Fagard R, Bjørnstad HH, Anastassakis A, Arbustini E, Assanelli D (2005). Recommendations for competitive sports participation in athletes with cardiovascular disease: A consensus document from the Study Group of Sports Cardiology of the Working Group of Cardiac Rehabilitation and Exercise Physiology and the Working Group of Myocardial and Pericardial Diseases of the European Society of Cardiology. European Heart Journal.

[4] Maron BJ, Udelson JE, Bonow RO, Nishimura RA, Ackerman MJ, Estes NAM (2015). Eligibility and Disqualification Recommendations for Competitive Athletes With Cardiovascular Abnormalities: Task Force 3: Hypertrophic Cardiomyopathy, Arrhythmogenic Right Ventricular Cardiomyopathy and Other Cardiomyopathies, and Myocarditis: A Scientific Statement From the American Heart Association and American College of Cardiology. Journal of the American College of Cardiology.

[5] Caforio ALP, Pankuweit S, Arbustini E, Basso C, Gimeno-Blanes J, Felix SB (2013). Current state of knowledge on aetiology, diagnosis, management, and therapy of myocarditis: A position statement of the European Society of Cardiology Working Group on myocardial and pericardial diseases. European Heart Journal.

